# Pericardial effusion after definitive concurrent chemotherapy and intensity modulated radiotherapy for esophageal cancer

**DOI:** 10.1186/s13014-020-01498-3

**Published:** 2020-02-27

**Authors:** Tzu-Hui Pao, Wei-Lun Chang, Nai-Jung Chiang, Chia-Ying Lin, Wu-Wei Lai, Yau-Lin Tseng, Forn-Chia Lin

**Affiliations:** 1 Department of Radiation Oncology, National Cheng Kung University Hospital, College of Medicine, National Cheng Kung University, No.138, Sheng Li Road, Tainan, 70456 Taiwan; 2 Department of Internal Medicine, National Cheng Kung University Hospital, College of Medicine, National Cheng Kung University, Tainan, Taiwan; 3 National Institute of Cancer Research, National Health Research Institutes, Tainan, Taiwan; 4 Department of Diagnostic Radiology, National Cheng Kung University Hospital, College of Medicine, National Cheng Kung University, Tainan, Taiwan; 5 Department of Surgery, National Cheng Kung University Hospital, College of Medicine, National Cheng Kung University, Tainan, Taiwan

**Keywords:** Esophageal cancer, Chemoradiotherapy, Intensity modulated radiotherapy, Pericardial effusion

## Abstract

**Background:**

The literature regarding pericardial effusion after definitive concurrent chemotherapy and intensity modulated radiotherapy (IMRT) for esophageal cancer was lacking. This study aimed to investigate the risk factors of pericardial effusion in esophageal cancer patients undergoing definitive concurrent chemotherapy and IMRT.

**Methods:**

A total of 126 consecutive esophageal cancer patients treated with definitive concurrent chemotherapy and IMRT between 2008 and 2018 were reviewed. The pericardial effusion was determined on computed tomography scan of the chest and graded by the Common Terminology Criteria for Adverse Events, version 4.0. The cumulative incidence of pericardial effusion was estimated by the Kaplan–Meier method and compared between groups by the log-rank test. The risk factors of pericardial effusion were determined with multivariate Cox proportional hazards regression analysis.

**Results:**

The median follow-up time was 14.0 months. Thirty-seven (29.4%) patients had pericardial effusion after a median interval of 6.6 months since the end of IMRT. The cumulative incidence of pericardial effusion of any grade was higher in patients with mean heart dose > 23.45 Gy (*p* = 0.00018), heart V30 > 33.55% (*p* = 0.00015), mean pericardium dose > 20.33 Gy (*p* = 0.00027), and pericardium V20 > 42.55% (p = 0.00018). Furthermore, eight (6.3%) patients had symptoms related to pericardial effusion and were considered as cases with pericardial effusion ≥ grade 3. The cumulative incidence of pericardial effusion ≥ grade 3 was higher in patients with pericardium V30 > 65.80% (*p* = 0.00028), V40 > 55.35% (*p* < 0.0001), and V60 > 24.70% (*p* = 0.0021). Multivariate analyses showed the above dose-volume parameters predicted the risk of pericardial effusion in esophageal cancer.

**Conclusions:**

Dose-volume parameters predicting the risk of pericardial effusion were identified in esophageal cancer treated with definitive concurrent chemotherapy and IMRT. They could be applied as constraints of IMRT for esophageal cancer.

## Introduction

Esophageal cancer is the sixth leading cause of cancer-related death globally [1]. Definitive concurrent chemoradiotherapy (CCRT) is the standard care for locally advanced esophageal cancer [2–4]. Aside from survival and disease control, treatment adverse events are concerns in patients undergoing CCRT.

Pericardial effusion has been recognized as the most common late cardiac toxicity in esophageal cancer patients treated by CCRT [5, 6]. According to retrospective studies in which three-dimensional conformal radiotherapy (3DCRT) was utilized with concurrent chemotherapy for esophageal cancer [7–10], the prevalence of pericardial effusion after CCRT was 27.7–52.2%. Grade 3 or higher pericardial effusion, which was considered clinically more important, was observed in 3.7–16% patients [5, 7, 9, 11–13]. However, the prevalence and risk factors of pericardial effusion in esophageal cancer patients receiving concurrent chemotherapy and intensity modulated radiotherapy (IMRT) remain largely unknown.

In this study, we analyzed a single-institution cohort of esophageal cancer patients treated by definitive CCRT with IMRT technique. The prevalence and predictors of pericardial effusion were investigated. Moreover, we reported the clinical course of pericardial effusion which was less depicted in the literature.

## Methods

### Patients and study design

This study was approved by the institutional review board of our hospital. Patients with primary esophageal cancer treated by definitive CCRT at our institution between 2008 and 2018 were reviewed. They were recruited on the basis of criteria as follows: newly pathologically confirmed esophageal cancer without distant metastasis, no past history of thoracic radiotherapy, no pericardial effusion before CCRT, CCRT via IMRT and conventional fractionation with dose ≥ 50 Gy, and follow-up after CCRT ≥ 3 months. Pre-existing cardio-pulmonary diseases other than pericardial effusion did not serve as selection criteria of patients in the current research. The pre-treatment evaluation of esophageal cancer included esophagogastroduodenoscopy, endoscopic ultrasonography, computed tomography (CT) of the chest and abdomen, and bone scan. The clinical stage was classified according to the seventh edition of the American Joint Committee on Cancer staging system.

### Definitive concurrent chemoradiotherapy

All patients received a standard definitive CCRT protocol for esophageal cancer with IMRT technique. The simulation CT scan was acquired at 5 mm slice thickness and transferred to Eclipse treatment planning system (Varian Medical Systems). The gross tumor volume (GTV) consisted of GTV of the primary (GTVp) and GTV of lymph nodes (GTVn). The clinical target volume (CTV) 1 included GTVp with a 5-cm craniocaudal and 1-cm radial margin along the esophagus, and GTVn with a 1-cm margin. The CTV 2 included GTVp with a 2-cm craniocaudal and 1-cm radial margin along the esophagus, and GTVn with a 1-cm margin. Representative images of target volume delineation were shown (Additional file 1: Fig. S1). The planning target volume (PTV) was generated by expanding 1 cm around the GTV and CTV in all directions. Radiotherapy was performed with sliding window IMRT at fixed gantry angles. A daily dose of 1.8–2 Gy was used with five fractions per week through 6 or 10-MV photons from a linear accelerator. CTV 1 and CTV 2 with the relevant PTV were sequentially treated to 36 and 50–50.4 Gy, respectively. Thereafter, GTV with the relevant PTV was boosted up to 66–66.6 Gy if dose constraints of the organs at risk could be met. Normal tissue-dose constraints included spinal cord (50 Gy to 5 cm), heart (50 Gy to one-third of the heart volume, V50 < 33%), lung (20 Gy to one-third of the lung volume, V20 < 33%), stomach (55 Gy to any part of the stomach volume, D_max_ < 50 Gy), and liver (35 Gy to one-half of the liver volume, V35 < 50%). During radiation treatment, concurrent chemotherapy and supportive therapy were given.

### Dosimetric analysis

The heart was delineated manually on each axial slice of simulation CT scan. The superior aspect began from the level of the inferior border of the pulmonary artery passing the midline and extended inferiorly to the cardiac apex [14]. The pericardium was defined according to the RTOG Contouring Atlases for Organs at Risk in Thoracic Radiation Therapy [15]**,** and was generally a sac with a 3-mm thickness around the heart and the root of great vessels [16]. Dose volume histogram of the heart and pericardium were subsequently generated using the treatment planning system. We calculated the following dose-volume parameters of the heart or pericardium: maximal dose, mean dose, and the percent volumes receiving doses ≥ 5 Gy (V5), ≥ 10 Gy (V10), ≥ 20 Gy (V20), ≥ 30 Gy (V30), ≥ 40 Gy (V40), ≥ 50 Gy (V50), and ≥ 60 Gy (V60).

### Evaluation of pericardial effusion

Follow-up evaluations included clinical examinations, esophagogastroduodenoscopy, and CT scan of the chest at 1 month after CCRT and then every 3–6 months. In addition, electrocardiography, echocardiogram, and other cardiovascular evaluations were arranged as clinically indicated. The pericardial effusion was determined on CT scan of the chest and graded by the Common Terminology Criteria for Adverse Events, version 4.0. Accordingly, symptomatic effusion was defined as effusion ≥ grade 3. To elucidate the clinical course of pericardial effusion, clinical symptoms and signs, CT images, electrocardiograms, echocardiograms, managements for pericardial effusion, and outcomes were reviewed.

### Statistical analysis

The data cutoff date was June 26, 2019. The time to development of pericardial effusion was defined as the interval from the end of IMRT to the first identification of pericardial effusion. Patients without pericardial effusion were censored at the last follow-up or death. The cumulative incidence of pericardial effusion was estimated by the Kaplan–Meier method and compared between groups by the log-rank test. More specifically, the optimal cut-off value of each dose-volume parameter was selected based on the receiver operating characteristic curve and Youden’s index. The variables that showed a trend in univariate analysis (*p* < 0.1) were used in a multivariate Cox proportional hazards regression analysis. A *p*-value < 0.05 was considered statistically significant. Statistical analyses were performed with SPSS version 22.0 software and R version 3.5.1 for Windows.

## Results

### Characteristics of the enrolled patients

Of the 204 patients reviewed, 126 patients matched the recruitment criteria while 78 patients were excluded from the analysis with reasons as follows: stage IV (*n* = 21), radiation dose < 50 Gy (*n* = 23), post-CCRT follow-up < 3 months (*n* = 32), and use of 3DCRT technique (n = 23). Table 1 summarized demographic and clinical characteristics of the enrolled 126 patients. Seven (5.6%) patients had a history of cardiovascular disease (2 coronary artery disease, 3 congestive heart failure, 1 aortic valve infectious endocarditis after valve replacement, and 1 arrhythmia). The median radiation dose was 61.2 Gy (range, 50–66.6 Gy). Fluoropyrimidine-based chemotherapy regimens were used in 118 (93.7%) patients. Most patients received either cisplatin (25 mg/m^2^) plus fluorouracil (1000 mg/m^2^) given intravenously every week or cisplatin (20 mg/m^2^ daily, on day 1–4) plus fluorouracil (800 mg/m^2^ daily, on day 1–4) given intravenously every 4 weeks. Other regimens were utilized at the discretion of physicians (Additional file 2: Table S1). Furthermore, during CCRT, enteral nutrition support was given via nasogastric, percutaneous endoscopic gastrostomy, and feeding jejunostomy tubes in nine (7.1%), 11 (8.7%), and 17 (13.5%) patients, respectively. Medications for emesis or pain as well as intravenous hydration were given as clinically indicated.

**Table 1:** Demographic and Clinical Characteristics of Patients at Baseline

Characteristic	No. of patients (%)	Univariate analysis^a^*P* value
Age (years)		
Median (Range)	56.5 (34–81)	
≤ 56: > 56	63 (50): 63 (50)	.293
Gender		
Male: Female	121 (96.0): 5 (4.0)	.077
Body mass index		
Median (Range)	21.4 (15.5–30.0)	
≤ 21.4: > 21.4	64 (50.8): 62 (49.2)	.721
Body surface area		
Median (Range)	1.65 (1.3–2.1)	
≤ 1.65: > 1.65	66 (52.4): 60 (47.6)	.784
Eastern Cooperative Oncology Group performance status		
0: 1: 2: 3	12 (9.5): 98 (77.8): 15 (11.9): 1 (0.8)	.539
Stage		
I: II: III	2 (1.6): 10 (7.9): 114 (90.5)	.600
Tumor location		
U: M: L	51 (40.5): 30 (23.8): 20 (15.9)	.001
U + M	9 (7.1)	
U + M + L	1 (0.8)	
M + L	15 (11.9)	
Histology		
Squamous cell carcinoma	121 (96.0)	.508
Adenocarcinoma	3 (2.4)	
Poorly differentiated carcinoma	2 (1.6)	
Smoking		
Yes: No	114 (90.5): 12 (9.5)	.350
Alcohol		
Yes: No	115 (91.3): 11 (8.7)	.094
Hypertension		
Yes: No	25 (19.8): 101 (80.2)	.418
Diabetes		
Yes: No	15 (11.9): 111 (88.1)	.582
Cardiovascular disease		
Yes: No	7 (5.6): 119 (94.4)	.097
Radiation dose (Gray)		
Median (range)	61.2 (50–66.6)	
≤ 60: > 60	52 (41.3): 74 (58.7)	.238
Chemotherapy regimen		
Fluoropyrimidine-based	118 (93.7)	.900
Taxane-based	4 (3.2)	
Others	4 (3.2)	

Abbreviations: *L* lower thoracic esophagus, *M* middle thoracic esophagus, *U* upper thoracic esophagus

^a^Univariate analysis of patients’ characteristics associated with pericardial effusion

### Clinical characteristics and dose-volume parameters associated with pericardial effusion of any grade

**Fig. 1:**
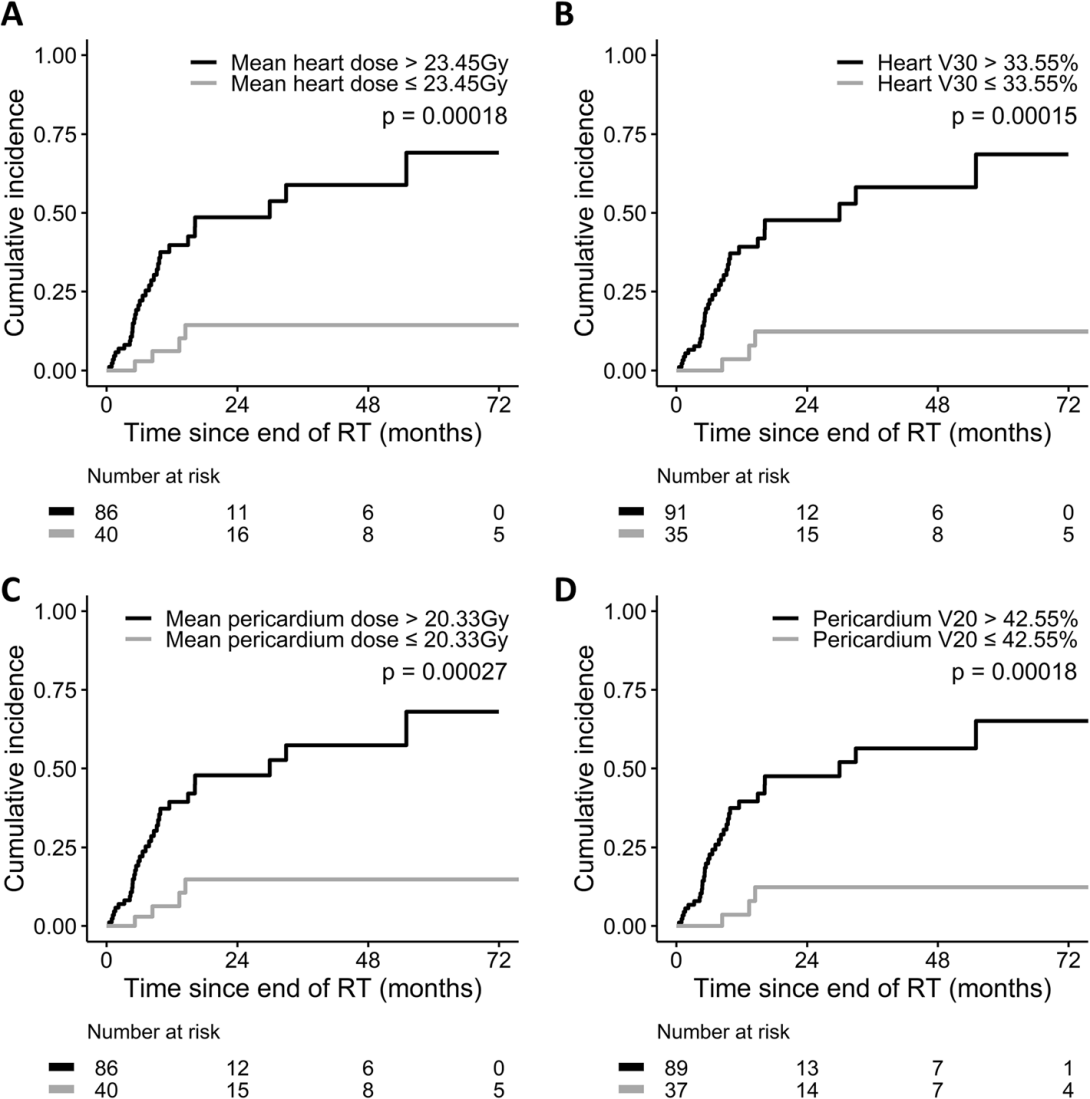
Cumulative incidence of pericardial effusion by **a** mean heart dose, **b** heart V30, **c** mean pericardium dose, and **d** pericardium V20

The median follow-up was 14.0 months (range, 3.1–107.7). Pericardial effusion was identified in 37 (29.4%) patients after a median interval of 6.6 months (range, 0.46–55.0) from the end of IMRT. Tumor location and 14 dose-volume parameters were associated with the development of pericardial effusion in univariate analyses (Tables 1 and 2). Body mass index, body surface area, and other clinicopathologic features did not correlate with the appearance of pericardial effusion (Table 1). In addition, the heart was in direct contact with CTV in 97 (77.0%) patients, thereby the distance between CTV and the heart being zero. For the remaining 29 cases, the distance between CTV and the heart was < 1 cm, 1–2 cm, and > 2 cm in 13 (10.3%), eight (6.3%), and eight (6.3%) patients, respectively. The distance between CTV and the heart was not associated with the development of pericardial effusion (*p* = 0.107). Multivariate analyses showed the dose-volume parameters were the independent risk factors for pericardial effusion of any grade (Table 3, Additional file 3: Table S2, and Additional file 4: Table S3). According to the values of hazard ratios (Table 3), we selected mean heart dose, heart V30, mean pericardium dose, and pericardium V20 as representative dose-volume parameters. The cumulative incidence of pericardial effusion of any grade was higher in patients with mean heart dose > 23.45 Gy (*p* = 0.00018, Fig. 1a), heart V30 > 33.55% (*p* = 0.00015, Fig. 1b), mean pericardium dose > 20.33 Gy (*p* = 0.00027, Fig. 1c), and pericardium V20 > 42.55% (p = 0.00018, Fig. 1d).

**Table 2:** Univariate Analysis of Dose-volume Variables Associated with Pericardial Effusion

Parameters	Pericardial effusion of any grade			Pericardial effusion ≥ Grade 3		
	Cutoff	*P* value	HR (95% CI)	Cutoff	*P* value	HR (95% CI)
Heart						
Maximal (Gy)	67.24	0.239	1.485 (0.769–2.870)	55.24	0.222	3.695 (0.453–30.128)
Mean (Gy)	23.45	0.001	5.876 (2.068–16.697)	30.99	0.133	81.645 (0.260–25,636.991)
V5 (%)	76.55	0.002	4.585 (1.776–11.835)	92.00	0.126	93.028 (0.281–30,852.401)
V10 (%)	68.15	0.001	4.739 (1.837–12.224)	84.20	0.134	81.108 (0.258–25,461.652)
V20 (%)	50.70	0.001	6.942 (2.118–22.753)	73.90	0.137	77.714 (0.251–24,062.690)
V30 (%)	33.55	0.001	7.235 (2.204–23.749)	57.45	0.130	85.917 (0.268–27,518.745)
V40 (%)	27.90	0.001	4.283 (1.864–9.841)	31.90	0.137	77.772 (0.251–24,088.957)
V50 (%)	10.55	0.002	3.797 (1.653–8.723)	10.95	0.161	60.587 (0.195–18,812.141)
V60 (%)	9.30	0.054	1.909 (0.988–3.690)	26.00	0.021	7.133 (1.343–37.891)
Pericardium						
Maximal (Gy)	55.14	0.198	1.673 (0.764–3.663)	53.97	0.406	27.314 (0.011–66,655.501)
Mean (Gy)	20.33	0.001	5.623 (1.981–15.958)	30.18	0.131	85.491 (0.267–27,370.316)
V5 (%)	84.50	0.001	3.758 (1.709–8.262)	89.15	0.128	89.096 (0.275–28,826.399)
V10 (%)	48.50	0.003	6.135 (1.876–20.066)	82.65	0.116	119.578 (0.305–46,932.571)
V20 (%)	42.55	0.001	6.997 (2.141–22.863)	71.55	0.120	109.854 (0.295–40,886.708)
V30 (%)	33.35	0.001	5.569 (1.961–15.817)	65.80	0.008	17.537 (2.137–143.927)
V40 (%)	28.90	< 0.001	4.120 (1.868–9.086)	55.35	< 0.001	19.316 (3.711–100.530)
V50 (%)	21.45	< 0.001	4.262 (2.086–8.708)	16.10	0.151	66.216 (0.215–20,359.365)
V60 (%)	11.10	0.066	1.838 (0.961–3.514)	24.70	0.011	9.324 (1.676–51.885)

Abbreviations: *Gy* gray, *Vx* percentage of the heart or pericardium volume receiving more than x gray

**Table 3:** Multivariate Analysis of Dose-volume Variables Associated with Pericardial Effusion

Parameters	Pericardial effusion of any grade		Pericardial effusion ≥ Grade 3	
	*P* value	HR (95% CI)	*P* value	HR (95% CI)
Heart				
Mean (Gy)	< 0.001	9.792 (2.930–32.724)	NA	NA
V5 (%)	0.001	6.670 (2.172–20.481)	NA	NA
V10 (%)	0.001	7.292 (2.370–22.439)	NA	NA
V20 (%)	< 0.001	10.681 (2.844–40.111)	NA	NA
V30 (%)	< 0.001	10.813 (2.897–40.360)	NA	NA
V40 (%)	< 0.001	7.191 (2.487–20.796)	NA	NA
V50 (%)	0.002	4.977 (1.790–13.842)	NA	NA
V60 (%)	0.363	1.407 (0.674–2.937)	0.055	5.495 (0.967–31.234)
Pericardium				
Mean (Gy)	< 0.001	9.597 (2.850–32.310)	NA	NA
V5 (%)	0.001	6.408 (2.194–18.713)	NA	NA
V10 (%)	0.001	8.507 (2.271–31.863)	NA	NA
V20 (%)	< 0.001	10.324 (2.780–38.343)	NA	NA
V30 (%)	0.001	8.269 (2.503–27.319)	0.010	32.309 (2.311–451.777)
V40 (%)	< 0.001	7.064 (2.430–20.540)	0.005	16.715 (2.307–121.093)
V50 (%)	0.001	5.312 (1.977–14.274)	NA	NA
V60 (%)	0.349	1.406 (0.689–2.868)	0.025	7.545 (1.293–44.011)

Variables taken into account in multivariate analysis included gender, tumor location, use of alcohol, cardiovascular disease and one of dose-volume parameters

Abbreviations: *Gy* gray, *NA* not applicable, *Vx* percentage of the heart or pericardium volume receiving more than x gray

### Dose-volume parameters associated with grade 3 or higher pericardial effusion

**Fig. 2:**
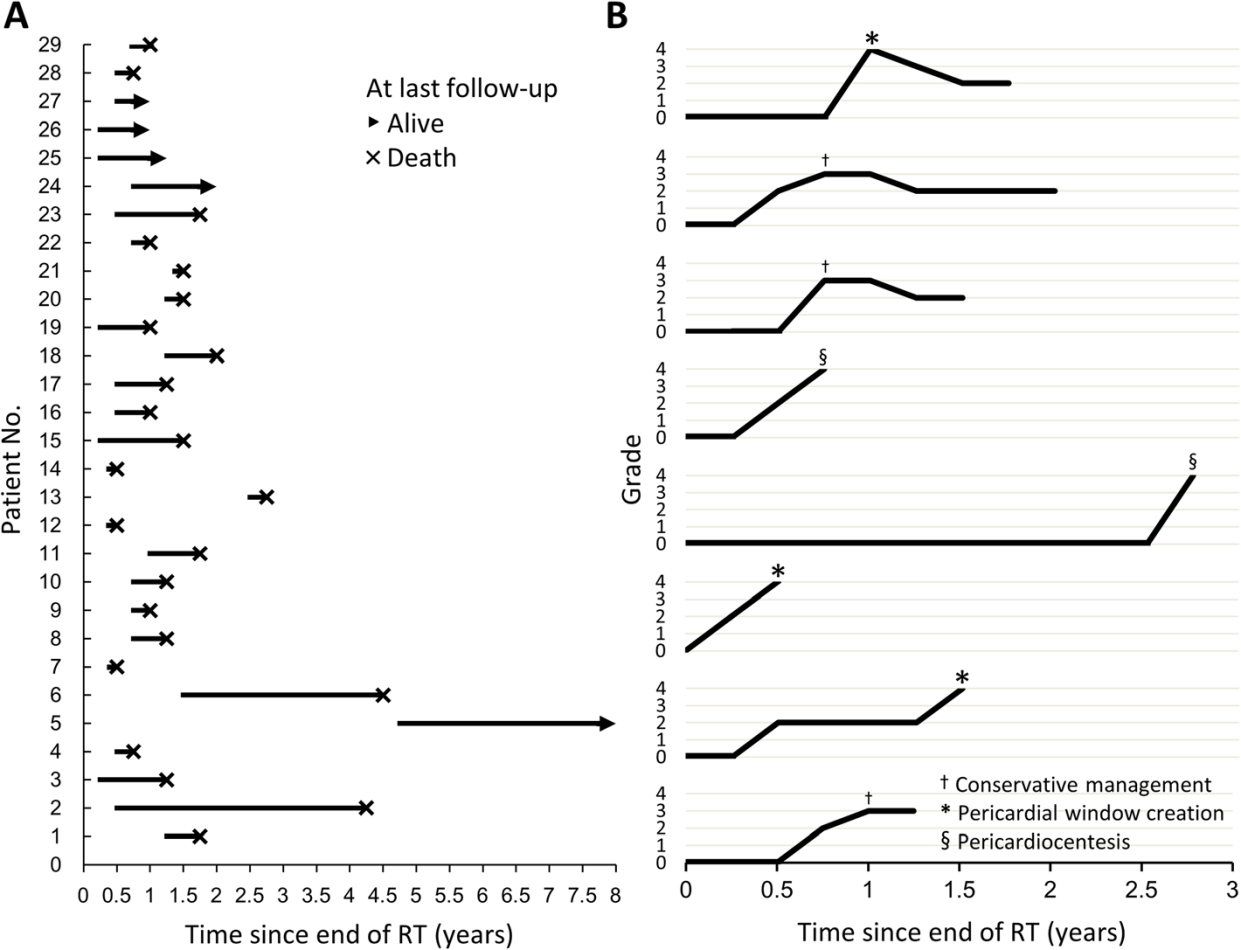
Clinical course of pericardial effusion in **a** 29 patients with grade 2 pericardial effusion, and **b** 8 patients with grade 3 or higher pericardial effusion

**Fig. 3:**
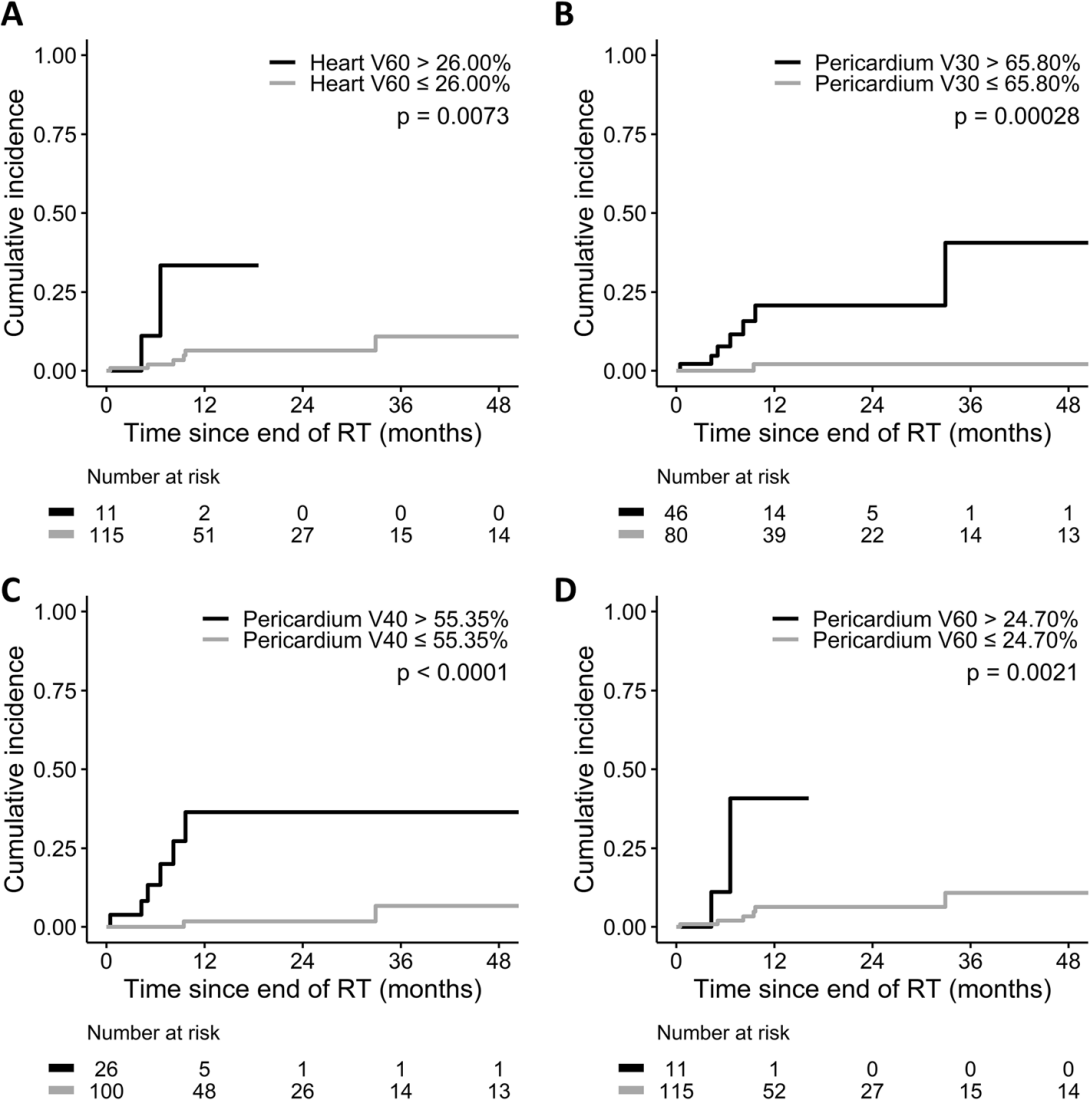
Cumulative incidence of grade 3 or higher pericardial effusion by **a** heart V60, **b** pericardium V30, **c** pericardium V40, and **d** pericardium V60

Among 37 patients with pericardial effusion, 29 patients were asymptomatic during follow-up. Eight patients had symptoms related to pericardial effusion after a median interval of 7.4 months (range, 0.46–32.92) from the end of IMRT and were regarded as cases with pericardial effusion ≥ grade 3 (Fig. 2). The cumulative incidence of pericardial effusion ≥ grade 3 was higher in patients with heart V60 > 26.00% (*p* = 0.0073, Fig. 3a), pericardium V30 > 65.80% (*p* = 0.00028, Fig. 3b), pericardium V40 > 55.35% (*p* < 0.0001, Fig. 3c), and pericardium V60 > 24.70% (*p* = 0.0021, Fig. 3d). Multivariate analyses showed pericardium V30, V40, and V60 were independent predictors of pericardial effusion ≥ grade 3 (Table 3 and Additional file 5: Table S4).

### Clinical course of pericardial effusion

Figure 2b showed the clinical course of pericardial effusion ≥ grade 3. All patients with pericardial effusion ≥ grade 3 presented with dyspnea. Four patients also had orthopnea, lower limbs edema, palpitation, or chest pain (Additional file 6: Table S5). Representative images of pericardial effusion were shown in Additional file 7: Fig. S2. The symptoms were considered related to pericardial effusion as the onset of the symptoms coincided with the development or increase of pericardial effusion. In addition, large pericardial effusion with right sided cardiac chamber collapse was disclosed on echocardiograms in six of eight symptomatic patients (Additional file 6: Table S5). These findings of echocardiograms further supported that the symptoms were related to pericardial effusion. On the other hand, with regard to the symptoms, etiologies other than pericardial effusion were also taken into considerations, properly evaluated, and individually managed. For example, pleural effusion was noted in five of eight symptomatic patients and considered as a possible coexisting cause of dyspnea (Additional file 6: Table S5). However, the presence of pleural effusion and other potential etiologies did not exclude the significant contribution of pericardial effusion to the symptoms. Accordingly, we recorded the pleural effusion of eight symptomatic patients as an adverse event ≥ grade 3. Furthermore, the symptoms were relieved after pericardial window and conservative treatment in one and two patients, respectively. But dyspnea persisted after conservative management in one, pericardiocentesis in two, and pericardial window in two patients. All patients with pericardial effusion ≥ grade 3 died at data cutoff date, due to oropharyngeal cancer in one and esophageal cancer progression in seven patients.

## Discussion

The present study analyzed 126 esophageal cancer patients undergoing definitive CCRT with IMRT technique. Pericardial effusion of any grade developed in 37 (29.4%) patients after a median interval of 6.6 months from the end of CCRT. Among them, eight patients became symptomatic during follow-up and were regarded as cases with pericardial effusion ≥ grade 3. Dose-volume parameters of heart and pericardium influencing the cumulative incidence of pericardial effusion were identified by multivariate analyses.

Prior studies have reported pericardial effusion after CCRT for esophageal cancer [5, 7–10, 12, 13, 16]. Notably, several key factors differentiated our data from previously published ones. To begin with, IMRT was utilized in the present study while 3DCRT was used in the previous researches. To the best of authors’ knowledge, we were the first to report the pericardial effusion in esophageal cancer patients treated by definitive concurrent chemotherapy and IMRT. In terms of the prevalence of pericardial effusion, IMRT was comparable to 3DCRT. In addition, previous studies examined the risk factors of either pericardial effusion of any grade or those ≥ grade 3. More comprehensively, the present study investigated factors independently influencing the cumulative incidence of both pericardial effusion of any grade and those ≥ grade 3 by multivariate analyses. The identified risk factors in the current study were similar to some of those found in the published 3DCRT cohorts. Finally, the clinical course of pericardial effusion was reported in the present study. The longitudinal change of pericardial effusion in individual patients could be more clearly viewed with the time scale.

The prevalence of pericardial effusion of any grade in the current cohort was 29.4% which was comparable to 27.7–52.2% in the literature using 3DCRT [7–10]. These data indicated that the prevalence of pericardial effusion was not reduced by IMRT when compared to 3DCRT. In line with previously published results, we found some dose-volume parameters of heart and pericardium were the independent predictors of pericardial effusion after CCRT in esophageal cancer. For example, pericardium V30 > 46% and V20 were identified as the important parameters associated with pericardial effusion of any grade by other groups [8, 16]. In the present study, pericardium V30 > 33.35% and V20 > 42.55% correlated with the higher cumulative incidence of pericardial effusion of any grade. However, there is no consensus about which dose-volume parameter of heart or pericardium is the most reliable to predict the risk of pericardial effusion after CCRT in esophageal cancer [7–10, 16]. Based on the values of hazard ratios in multivariate analyses, we suggested mean heart dose ≤23.45 Gy, heart V30 ≤ 33.55%, mean pericardium dose ≤20.33 Gy, and pericardium V20 ≤ 42.55% as representative dose-volume constraints to reduce the risk of pericardial effusion after CCRT in esophageal cancer. On the other side, whether clinical or demographic factors served as risk factors for pericardial effusion in esophageal cancer patients receiving CCRT remained controversial [8, 10]. Our data did not support that clinical and demographic factors significantly influenced the risk of pericardial effusion after CCRT in esophageal cancer.

In the present study, the prevalence of pericardial effusion ≥ grade 3 was 6.3% and seemed not decreased by IMRT technique when compared to 3.7–16% in the cohorts using 3DCRT [5, 7, 9, 11–13]. Three studies investigated independent risk factors affecting symptomatic radiation-induced cardiac disease in esophageal cancer patients. However, the identified factors were not specific for pericardial effusion [5, 9, 11]. On the other hand, Fukada et al. reported that pericardium mean dose > 36.5 Gy and V45 > 58% were the risk factors of pericardial effusion ≥ grade 3 after CCRT for esophageal cancer [7]. In the present study, multivariate analyses showed pericardium V30 > 65.8%, V40 > 55.35%, and V60 > 24.7% served as risk factors for pericardial effusion ≥ grade 3 after CCRT in esophageal cancer. Obviously, pericardium V40 > 55.35% identified by our group was quite similar to pericardium V45 > 58% reported by Fukada et al. Therefore, pericardium V40 or V45 with a cutoff value of around 55–58% could be a suitable dose-volume parameter for predicting the risk of pericardial effusion ≥ grade 3 after CCRT in esophageal cancer. Further validation with independent cohorts is warranted.

Cardiotoxicity has also been noted in patients with non-small cell lung cancer (NSCLC) treated by CCRT. Pericardial effusion was the most common cardiac event [17, 18]. The prevalence of pericardial effusion in NSCLC was 15.2–24.2%, which was similar to that in esophageal cancer treated with CCRT. In NSCLC, some radiation dosimetric parameters were found to be prognostic factors for overall cardiac events including acute coronary syndrome, congestive heart failure, arrhythmia, cardiac arrest, valvular disease, pericardial effusion, and pericarditis, but not specific for pericardial effusion. To study risk factors of the specific cardiac adverse event is warranted in the future. In addition, when compared to 3DCRT, IMRT produced lower heart doses in NSCLC treated with CCRT [19]. However, whether IMRT reduced cardiac exposure to radiation in esophageal cancer could not be answered in the present research as all our patients were treated with IMRT. It would be interesting to conduct a study comparing the cardiac radiation dose between 3DCRT and IMRT cohorts.

Our study was limited by its retrospective research design and all potential inherent biases. In addition, we did dosimetric analyses based on planning CT scan and without consideration of cardiac physiological motion. Errors of estimation would exist under such circumstance but seemed unable to be corrected to date. Moreover, heterogeneous results among our and other similar studies possibly in part derived from variations in statistical methods, contouring definition of cardiac structures, and grading criteria of pericardial effusion. Consensus on study methodology and intergroup validations are suggested.

## Conclusions

We were the first to report the dose-volume parameters predicting the risk of pericardial effusion in esophageal cancer patients undergoing definitive concurrent chemotherapy and IMRT. After external validations, the identified parameters could be applied as constraints of IMRT in esophageal cancer.

## Supplementary information

**Additional file 1.** Figure S1. Representative images of target volume delineation on (a) axial, (b) sagittal, and (c) coronal plane of simulation CT scan. GTVp (green), GTVn (yellow), CTV1 (red), and CTV2 (orange).

**Additional file 2.** Table S1. Summary of the Chemotherapy Regimens

**Additional file 3.** Table S2. Multivariate Analysis of Clinical and Heart Dose-volume Variables Associated with Pericardial Effusion of Any Grade

**Additional file 4.** Table S3. Multivariate Analysis of Clinical and Pericardium Dose-volume Variables Associated with Pericardial Effusion of Any Grade

**Additional file 5.** Table S4. Multivariate Analysis of Clinical and Dose-volume Variables Associated with Pericardial Effusion ≥ Grade 3

**Additional file 6.** Table S5. Clinical Information of 8 Patients with Pericardial Effusion ≥ Grade 3

**Additional file 7.** Figure S2. Representative images of pericardial effusion ≥ Grade 3 on (a) axial and (b) coronal plane of CT scan

## Data Availability

The datasets used and/or analyzed during the current study are available from the corresponding author on reasonable request.

## Abbreviations

3DCRT: Three-dimensional conformal radiotherapy
CCRT: Concurrent chemoradiotherapy
CT: Computed tomography
CTV: Clinical target volume
GTV: Gross tumor volume
GTVn: Gross tumor volume of lymph nodes
GTVp: Gross tumor volume of the primary
IMRT: Intensity modulated radiotherapy
NSCLC: Non-small cell lung cancer
PTV: Planning target volume
