# Supplementary figures and images for: Pericardial effusion after definitive concurrent chemotherapy and intensity modulated radiotherapy for esophageal cancer
Source: Radiat Oncol. 2020 Feb 27;15:48. doi: 10.1186/s13014-020-01498-3 (PMC7045635; doi:10.1186/s13014-020-01498-3)

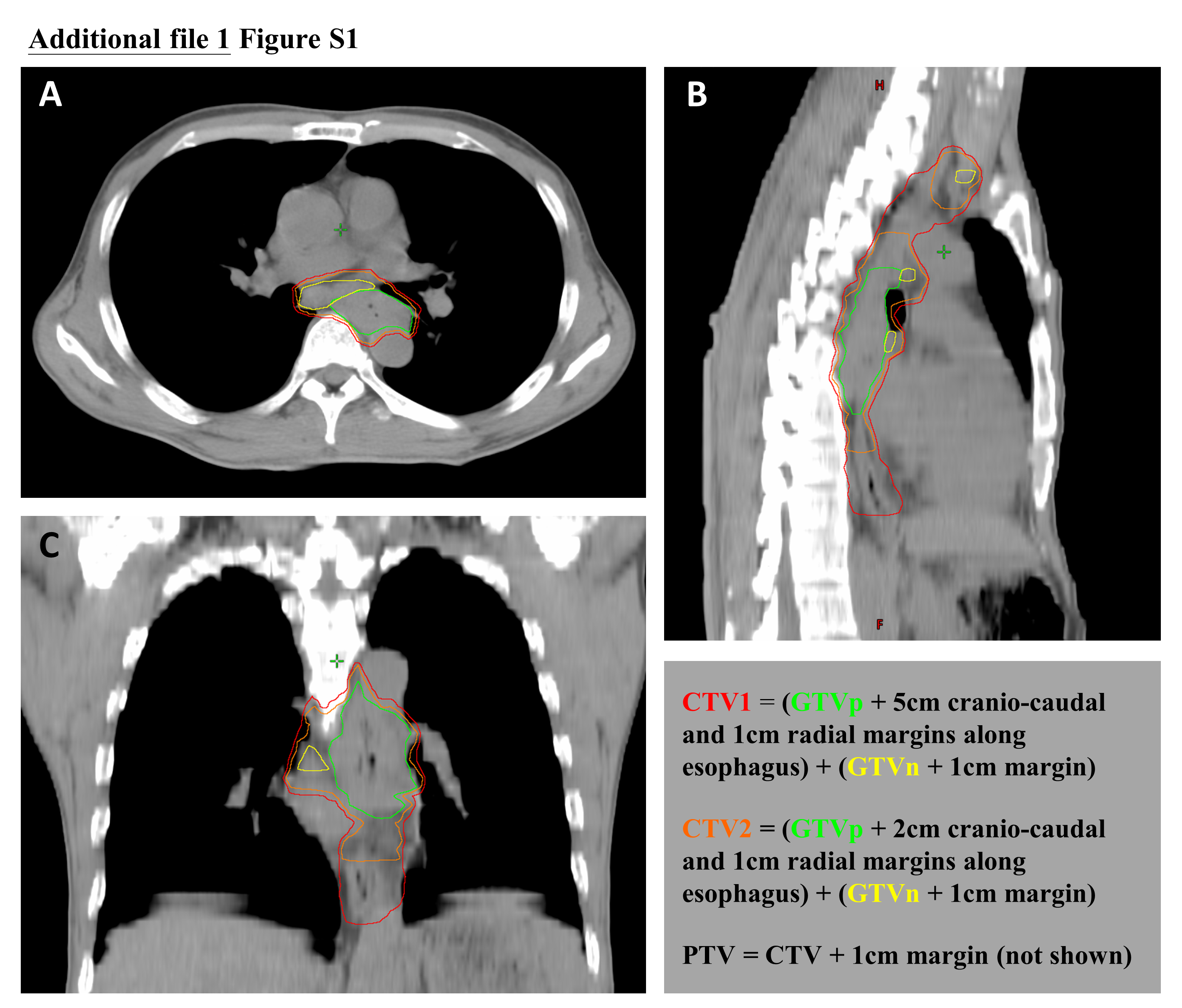

Supplement: Supplementary file 1 — Additional file 1. Figure S1. Representative images of target volume delineation on (a) axial, (b) sagittal, and (c) coronal plane of simulation CT scan. GTVp (green), GTVn (yellow), CTV1 (red), and CTV2 (orange). [file 13014_2020_1498_MOESM1_ESM.tif]

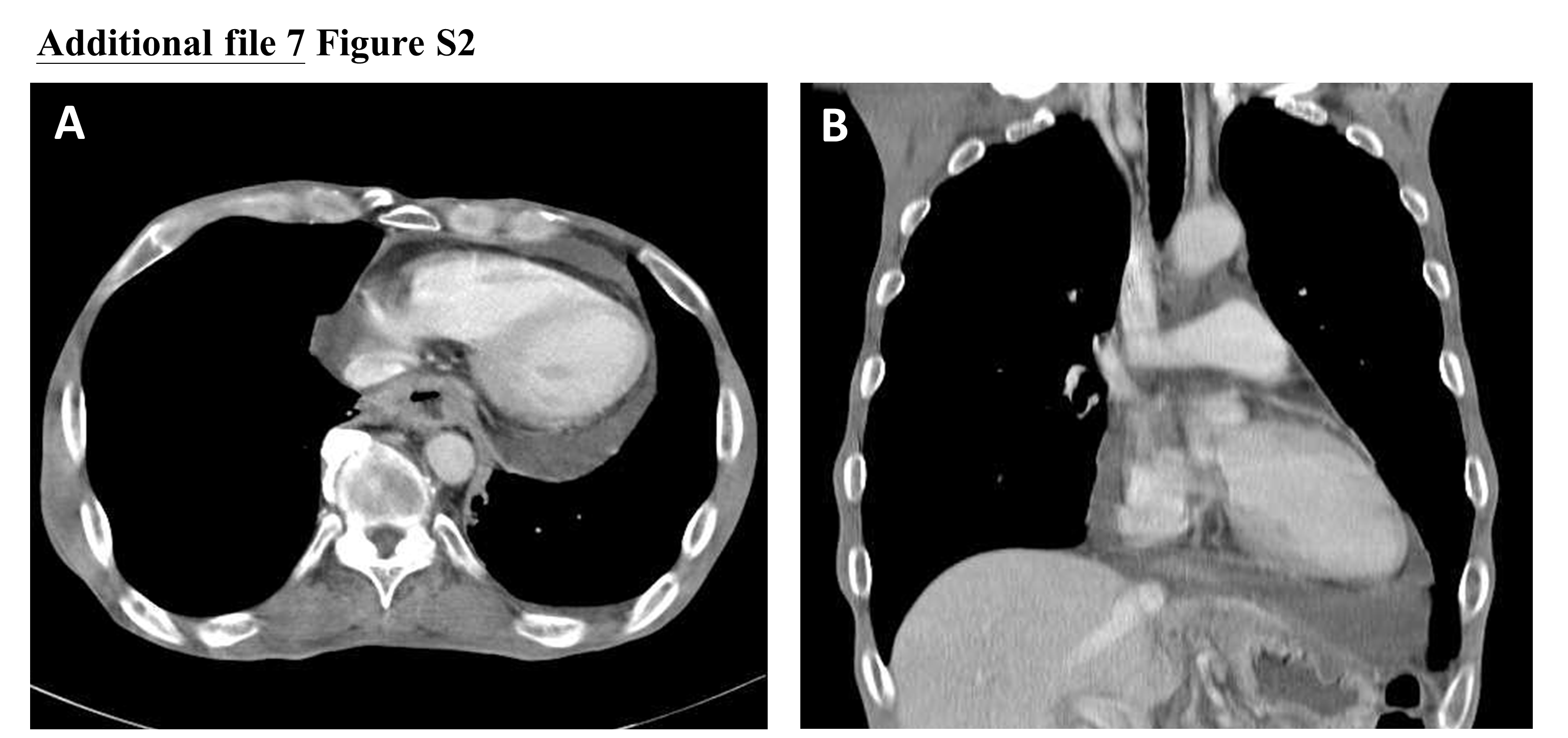

Supplement: Supplementary file 7 — Additional file 7. Figure S2. Representative images of pericardial effusion ≥ Grade 3 on (a) axial and (b) coronal plane of CT scan [file 13014_2020_1498_MOESM7_ESM.tif]
