# Supplementary material for: Pericardial effusion after definitive concurrent chemotherapy and intensity modulated radiotherapy for esophageal cancer
Source: Radiat Oncol. 2020 Feb 27;15:48. doi: 10.1186/s13014-020-01498-3 (PMC7045635; doi:10.1186/s13014-020-01498-3)
Supplement: Supplementary file 2 — Additional file 2. Table S1. Summary of the Chemotherapy Regimens [file 13014_2020_1498_MOESM2_ESM.pdf]

Additional file 2

Table S1. Summary of the Chemotherapy Regimens

**Chemotherapy regimen**	**Number (%)**
P (25 mg/m^2) + F (1000 mg/m^2) QW	61 (48.4)
P (20 mg/m^2 daily, D1–4) + F (800 mg/m^2 daily, D1–4) Q4W	31 (24.6)
P (25-40 mg/m^2) + F (2000-2600 mg/m^2) + L (200-300 mg/m^2) Q2W	9 (7.1)
P (20-25 mg/m^2) + F (1500-2000 mg/m^2) + L (150-200 mg/m^2) QW	9 (7.1)
P (25 mg/m^2) + F (2000 mg/m^2) + L (200 mg/m^2) + Cet (400 → 250 mg/m^2) QW	1 (0.8)
P (20 mg/m^2) + F (1600 mg/m^2) + L (160 mg/m^2) + E (30 mg/m^2) QW	1 (0.8)
P (25 mg/m^2) + F (2000 mg/m^2) + Epirubicin (30 mg/m^2) Q2W	1 (0.8)
F (1600 mg/m^2) QW	1 (0.8)
TS-1 (Tegafur/gimeracil/oteracil) 100 mg/day	1 (0.8)
F (2000 mg/m^2) + Carboplatin (AUC 4) Q2W	1 (0.8)
F (1600 mg/m^2) + L (160 mg/m^2) QW	1 (0.8)
F (2400 mg/m^2) + L (400 mg/m^2) + Oxaliplatin (85 mg/m^2) Q2W	1 (0.8)
T (80 mg/m^2) + P (15 mg/m^2) QW	2 (1.6)
T (50 mg/m^2) + Carboplatin (AUC 2) QW	1 (0.8)
T (35 mg/m^2) + P (15 mg/m^2) + Cet (400 → 250 mg/m^2) QW	1 (0.8)
P (40 mg/m^2) QW	2 (1.6)
Docetaxel (35 mg/m2) + P (35 mg/m^2) + F (1500 mg/m^2) Q2W	1 (0.8)
T (110 mg/m^2) + P (40 mg/m^2) + F (3000 mg/m^2) + L (400 mg/m^2) Q2W	1 (0.8)

Abbreviations: *Cet* cetuximab, *D* day, *E* etoposide, *F* fluorouracil, *L* leucovorin, *P* cisplatin, *QW* weekly, *Q2W* every 2 weeks, *Q4W* every 4 weeks, *T* paclitaxel
