# Supplementary material for: Pericardial effusion after definitive concurrent chemotherapy and intensity modulated radiotherapy for esophageal cancer
Source: Radiat Oncol. 2020 Feb 27;15:48. doi: 10.1186/s13014-020-01498-3 (PMC7045635; doi:10.1186/s13014-020-01498-3)
Supplement: Supplementary file 3 — Additional file 3. Table S2. Multivariate Analysis of Clinical and Heart Dose-volume Variables Associated with Pericardial Effusion of Any Grade [file 13014_2020_1498_MOESM3_ESM.pdf]

Additional file 3

Table S2. Multivariate Analysis of Clinical and Heart Dose-volume Variables Associated with Pericardial Effusion of Any Grade

	Variables	HR	95% CI	*P* value
**Model A**	Gender (female vs. male)	2.210	0.512-9.542	0.288
	Location (L/M vs. U)	1.647	0.752-3.607	0.212
	Alcohol (yes vs. no)	0.396	0.134-1.168	0.093
	Cardiovascular disease (yes vs. no)	0.000	0.000-.	0.975
	Mean heart dose (> 23.45 vs. ≤ 23.45 Gy)	9.792	2.930-32.724	<0.001
**Model B**	Gender (female vs. male)	1.780	0.442-7.168	0.418
	Location (L/M vs. U)	1.426	0.637-3.190	0.388
	Alcohol (yes vs. no)	0.381	0.135-1.080	0.069
	Cardiovascular disease (yes vs. no)	0.000	0.000-.	0.974
	Heart V5 (> 76.55 vs. ≤ 76.55%)	6.670	2.172-20.481	0.001
**Model C**	Gender (female vs. male)	1.770	0.442-7.088	0.420
	Location (L/M vs. U)	1.541	0.686-3.458	0.295
	Alcohol (yes vs. no)	0.382	0.136-1.075	0.068
	Cardiovascular disease (yes vs. no)	0.000	0.000-.	0.975
	Heart V10 (> 68.15 vs. ≤ 68.15%)	7.292	2.370-22.439	0.001
**Model D**	Gender (female vs. male)	1.913	0.473-7.735	0.363
	Location (L/M vs. U)	1.459	0.684-3.111	0.329
	Alcohol (yes vs. no)	0.345	0.121-0.981	0.046
	Cardiovascular disease (yes vs. no)	0.000	0.000-.	0.974
	Heart V20 (> 50.70 vs. ≤ 50.70%)	10.681	2.844-40.111	<0.001
**Model E**	Gender (female vs. male)	1.936	0.478-7.834	0.354
	Location (L/M vs. U)	1.425	0.670-3.030	0.358
	Alcohol (yes vs. no)	0.350	0.123-0.997	0.049
	Cardiovascular disease (yes vs. no)	0.000	0.000-.	0.974
	Heart V30 (> 33.55 vs. ≤ 33.55%)	10.813	2.897-40.360	<0.001
**Model F**	Gender (female vs. male)	1.701	0.432-6.697	0.447
	Location (L/M vs. U)	1.809	0.754-4.343	0.185
	Alcohol (yes vs. no)	0.441	0.159-1.224	0.116
	Cardiovascular disease (yes vs. no)	0.000	0.000-.	0.975
	Heart V40 (> 27.90 vs. ≤ 27.90%)	7.191	2.487-20.796	<0.001
**Model G**	Gender (female vs. male)	1.718	0.427-6.920	0.446
	Location (L/M vs. U)	1.310	0.564-3.045	0.530
	Alcohol (yes vs. no)	0.427	0.151-1.206	0.108

	Cardiovascular disease (yes vs. no)	0.000	0.000-.	0.975
	Heart V50 (> 10.55 vs. ≤ 10.55%)	4.977	1.790-13.842	0.002
**Model H**	Gender (female vs. male)	1.770	0.426-7.349	0.432
	Location (L/M vs. U)	1.725	0.794-3.746	0.168
	Alcohol (yes vs. no)	0.480	0.166-1.384	0.174
	Cardiovascular disease (yes vs. no)	0.000	0.000-.	0.976
	Heart V60 (> 9.30 vs. ≤ 9.30%)	1.407	0.674-2.937	0.363

Abbreviations: *Gy* gray, *L* lower thoracic esophagus, *M* middle thoracic esophagus, *U* upper thoracic esophagus, *Vx* percentage of the heart volume receiving more than x gray
