# Supplementary material for: Pericardial effusion after definitive concurrent chemotherapy and intensity modulated radiotherapy for esophageal cancer
Source: Radiat Oncol. 2020 Feb 27;15:48. doi: 10.1186/s13014-020-01498-3 (PMC7045635; doi:10.1186/s13014-020-01498-3)
Supplement: Supplementary file 4 — Additional file 4. Table S3. Multivariate Analysis of Clinical and Pericardium Dose-volume Variables Associated with Pericardial Effusion of Any Grade [file 13014_2020_1498_MOESM4_ESM.pdf]

Additional file 4

Table S3. Multivariate Analysis of Clinical and Pericardium Dose-volume Variables Associated with Pericardial Effusion of Any Grade

	Variables	HR	95% CI	*P* value
Model A	Gender (female vs. male)	2.190	0.507-9.464	0.294
	Location (L/M vs. U)	1.669	0.759-3.671	0.203
	Alcohol (yes vs. no)	0.390	0.132-1.152	0.088
	Cardiovascular disease (yes vs. no)	0.000	0.000-.	0.975
	Mean PC dose (> 20.33 vs. ≤ 20.33 Gy)	9.597	2.850-32.310	<0.001
Model B	Gender (female vs. male)	1.639	0.417-6.439	0.479
	Location (L/M vs. U)	1.882	0.738-4.803	0.186
	Alcohol (yes vs. no)	0.443	0.160-1.221	0.115
	Cardiovascular disease (yes vs. no)	0.000	0.000-.	0.974
	PC V5 (> 84.50 vs. ≤ 84.50%)	6.408	2.194-18.713	0.001
Model C	Gender (female vs. male)	1.898	0.464-7.761	0.373
	Location (L/M vs. U)	1.227	0.576-2.613	0.595
	Alcohol (yes vs. no)	0.339	0.118-0.973	0.044
	Cardiovascular disease (yes vs. no)	0.000	0.000-.	0.974
	PC V10 (> 48.50 vs. ≤ 48.50%)	8.507	2.271-31.863	0.001
Model D	Gender (female vs. male)	1.888	0.467-7.642	0.373
	Location (L/M vs. U)	1.387	0.652-2.950	0.395
	Alcohol (yes vs. no)	0.342	0.121-0.972	0.044
	Cardiovascular disease (yes vs. no)	0.000	0.000-.	0.974
	PC V20 (> 42.55 vs. ≤ 42.55%)	10.324	2.780-38.343	<0.001
Model E	Gender (female vs. male)	1.839	0.456-7.418	0.392
	Location (L/M vs. U)	1.441	0.662-3.140	0.358
	Alcohol (yes vs. no)	0.365	0.129-1.034	0.058
	Cardiovascular disease (yes vs. no)	0.000	0.000-.	0.974
	PC V30 (> 33.35 vs. ≤ 33.35%)	8.269	2.503-27.319	0.001
Model F	Gender (female vs. male)	1.657	0.424-6.476	0.468
	Location (L/M vs. U)	1.936	0.767-4.886	0.162
	Alcohol (yes vs. no)	0.462	0.168-1.275	0.136
	Cardiovascular disease (yes vs. no)	0.000	0.000-.	0.974
	PC V40 (> 28.90% vs. ≤ 28.90%)	7.064	2.430-20.540	<0.001
Model G	Gender (female vs. male)	1.573	0.402-6.151	0.515
	Location (L/M vs. U)	1.575	0.605-4.105	0.352
	Alcohol (yes vs. no)	0.527	0.192-1.449	0.215

	Cardiovascular disease (yes vs. no)	0.000	0.000-.	0.978
	PC V50 (> 21.45 vs. ≤ 21.45%)	5.312	1.977-14.274	0.001
**Model H**	Gender (female vs. male)	1.780	0.427-7.416	0.429
	Location (L/M vs. U)	1.739	0.811-3.725	0.155
	Alcohol (yes vs. no)	0.470	0.163-1.353	0.162
	Cardiovascular disease (yes vs. no)	0.000	0.000-.	0.976
	PC V60 (> 11.10 vs. ≤ 11.10%)	1.406	0.689-2.868	0.349

Abbreviations: *Gy* gray, *L* lower thoracic esophagus, *M* middle thoracic esophagus, *PC* pericardium, *U* upper thoracic esophagus, *Vx* percentage of the pericardium volume receiving more than x gray
