# Supplementary material for: Pericardial effusion after definitive concurrent chemotherapy and intensity modulated radiotherapy for esophageal cancer
Source: Radiat Oncol. 2020 Feb 27;15:48. doi: 10.1186/s13014-020-01498-3 (PMC7045635; doi:10.1186/s13014-020-01498-3)
Supplement: Supplementary file 5 — Additional file 5. Table S4. Multivariate Analysis of Clinical and Dose-volume Variables Associated with Pericardial Effusion ≥ Grade 3 [file 13014_2020_1498_MOESM5_ESM.pdf]

Additional file 5

Table S4. Multivariate Analysis of Clinical and Dose-volume Variables Associated with Pericardial Effusion ≥ Grade 3

	Variables	HR	95% CI	*P* value
**Model A**	Gender (female vs. male)	2.313	0.154-34.652	0.544
	Location (L/M vs. U)	6.803	0.775-59.712	0.084
	Alcohol (yes vs. no)	0.193	0.024-1.582	0.125
	Cardiovascular disease (yes vs. no)	0.000	0.000-.	0.990
	Heart V60 (> 26.00 vs. ≤ 26.00%)	5.495	0.967-31.234	0.055
**Model B**	Gender (female vs. male)	0.340	0.011-10.438	0.537
	Location (L/M vs. U)	1.367	0.073-25.712	0.835
	Alcohol (yes vs. no)	0.051	0.003-0.988	0.049
	Cardiovascular disease (yes vs. no)	0.000	0.000-.	0.990
	PC V30 (> 65.80 vs. ≤ 65.80%)	32.309	2.311-451.777	0.010
**Model C**	Gender (female vs. male)	0.440	0.020-9.898	0.605
	Location (L/M vs. U)	2.283	0.194-26.940	0.512
	Alcohol (yes vs. no)	0.097	0.008-1.143	0.064
	Cardiovascular disease (yes vs. no)	0.000	0.000-.	0.990
	PC V40 (> 55.35 vs. ≤ 55.35%)	16.715	2.307-121.093	0.005
**Model D**	Gender (female vs. male)	2.319	0.161-33.495	0.537
	Location (L/M vs. U)	7.144	0.814-62.723	0.076
	Alcohol (yes vs. no)	0.189	0.024-1.495	0.114
	Cardiovascular disease (yes vs. no)	0.000	0.000-.	0.991
	PC V60 (> 24.70 vs. ≤ 24.70%)	7.545	1.293-44.011	0.025

Abbreviations: *L* lower thoracic esophagus, *M* middle thoracic esophagus, *PC* pericardium, *U* upper thoracic esophagus, *Vx* percentage of the heart or pericardium volume receiving more than x gray
