# Supplementary material for: Pericardial effusion after definitive concurrent chemotherapy and intensity modulated radiotherapy for esophageal cancer
Source: Radiat Oncol. 2020 Feb 27;15:48. doi: 10.1186/s13014-020-01498-3 (PMC7045635; doi:10.1186/s13014-020-01498-3)
Supplement: Supplementary file 6 — Additional file 6. Table S5. Clinical Information of 8 Patients with Pericardial Effusion ≥ Grade 3 [file 13014_2020_1498_MOESM6_ESM.pdf]

Additional file 6

Table S5. Clinical Information of 8 Patients with Pericardial Effusion ≥ Grade 3

Patient	Symptoms	Electrocardiography	Echocardiography	Pleural effusion	Treatment
1	Dyspnea	Sinus rhythm, low voltage QRS complexes	Large pericardial effusion with RA and RV collapse	Bilateral	Pericardial window
2	Dyspnea	Not available	Thickened pericardium and pericardial effusion with RA collapse	Bilateral	Conservative management
3	Dyspnea	Sinus rhythm, low voltage in frontal leads	Pericardial effusion	Bilateral	Conservative management
4	Dyspnea, orthopnea	Sinus tachycardia, low voltage in frontal leads	Large pericardial effusion with RV collapse	Bilateral	Pericardiocentesis
5	Dyspnea, lower limbs edema	Atrial fibrillation, low voltage in frontal leads	Large pericardial effusion with RA and RV collapse	Left	Pericardiocentesis
6	Dyspnea, palpitation	Atrial fibrillation	Large pericardial effusion with RV collapse	No	Pericardial window
7	Dyspnea, chest pain	Sinus rhythm, low voltage in frontal leads	Large pericardial effusion with RV collapse	No	Pericardial window
8	Dyspnea	Sinus tachycardia	Large pericardial effusion	No	Conservative management

Abbreviations: *RA* right atrium, *RV* right ventricle
